# Functional Characterization of c-di-GMP Signaling-Related Genes in the Probiotic *Lactobacillus acidophilus*

**DOI:** 10.3389/fmicb.2018.01935

**Published:** 2018-08-29

**Authors:** Jiahui He, Wenhao Ruan, Jieli Sun, Fang Wang, Wenjuan Yan

**Affiliations:** ^1^Department of Stomatology, Nanfang Hospital, Southern Medical University, Guangzhou, China; ^2^Department of Neurobiology, School of Basic Medical Science, Southern Medical University, Guangzhou, China; ^3^Department of Stomatology, The Affiliated Shenzhen Maternity and Child Healthcare Hospital of the South Medical University, Shenzhen, China

**Keywords:** c-di-GMP signaling, *Lactobacillus acidophilus*, GGDEF domain, EAL domain, c-di-GMP receptor, exopolysaccharide

## Abstract

The bacterial second messenger cyclic diguanylate monophosphate (c-di-GMP) regulates a series of cellular functions, including biofilm formation, motility, virulence, and other processes. In this study, we confirmed the presence of several c-di-GMP related genes and evaluated their activities and functions in *Lactobacillus* species. Bioinformatic and biochemical analyses revealed that *Lactobacillus acidophilus* La-14 have an active c-di-GMP phosphodiesterase (PdeA) that may act in the metabolic cycle of c-di-GMP. A GGDEF protein (DgcA) induced two c-di-GMP-dependent phenotypes (low motility and high production of curli fimbriae) in *Escherichia coli* by heterologously expressed *in vivo* but showed no diguanylate cyclases activity *in vitro* while in the expression without the N-terminal transmembrane domain. The degenerated EAL-domain protein (PdeB), encoded by the last gene in the *gts* operon, serve as a c-di-GMP receptor which may be associated with exopolysaccharide (EPS) synthesis in *L. acidophilus*. Heterologously expressed GtsA and GtsB, encoded by the *gts* operon, stimulated EPS and biofilm formation in *E. coli* BL21. Constitutive expression in *L. acidophilus* revealed that a high concentration of intracellular DgcA levels increased EPS production in *L. acidophilus* and enhanced the co-aggregation ability with *E. coli* MG1655, which may be beneficial to the probiotic properties of *Lactobacillus* species. Our study imply that the c-di-GMP metabolism-related genes, in *L. acidophilus*, work jointly to regulate its functions in EPS formation and co-aggregation.

## Introduction

Cyclic diguanylate monophosphate (c-di-GMP), formed by the condensation of two GTP molecules, is a secondary messenger that is widely distributed in bacteria and is involved in the regulation of multiple bacterial physiological functions (Hengge, 2009). Opposing activities of diguanylate cyclases (DGCs) containing the GGDEF domain and phosphodiesterases (PDEs) containing EAL or HD-GYP domains control cellular c-di-GMP homeostasis (Römling et al., 2013). Genes encoding GGDEF and EAL protein families are distributed unevenly among the genomes of various species. For example, *Staphylococcus aureus* possesses only one GGDEF protein, GdpS, without DGC activity *in vitro* and is involved in virulence regulation through an RNA-dependent pathway (Holland et al., 2008; Römling et al., 2013). By comparison, more than 90 genes potentially encoding c-di-GMP-metabolizing enzymes were predicted in *Vibrio vulnificus* (Römling et al., 2013). These GGDEF or EAL domains, in tandem with other signaling domains and located in the cytoplasm or cytomembranes, precisely regulate local intracellular c-di-GMP concentrations by responding to diverse upstream activating signals. C-di-GMP regulates a variety of physiological processes, including cell-cell interactions (Matsuyama et al., 2016; Lin C. S. et al., 2017), biofilm formation and dispersal (Ha and O'Toole, 2015; Skariyachan et al., 2018), cell motility (Orr and Lee, 2016), and the responses to a variety of external stimulation, such as oxygen (Burns et al., 2016), nitric oxide (Rinaldo et al., 2018), and light (Blain-Hartung et al., 2017). The c-di-GMP signaling pathway is present in many Gram-negative bacteria but is less reported in Gram-positive bacteria (Purcell and Tamayo, 2016). In recent years, however, the existence of a c-di-GMP signaling pathway has also been confirmed in many Gram-positive bacteria, such as *Streptomyces coelicolor* (den Hengst et al., 2010), *Clostridium difficile* (Purcell et al., 2012), *Bacillus subtilis* (Gao et al., 2013), and *Listeria monocytogenes* (Chen et al., 2014). In these species, c-di-GMP signaling primarily regulates flagellum synthesis, production of adhesion factor in response to surface contact, and production of extracellular polymeric substances (Purcell and Tamayo, 2016; Bedrunka and Graumann, 2017a).

The recent discovery of c-di-GMP signaling in *Firmicutes* prompted us to focus on the species of *Lactobacillus*, especially *Lactobacillus acidophilus*. So far, the c-di-GMP-metabolizing enzymes in *Lactobacillus* have been poorly characterized except for a degenerated EAL-domain protein (Lp_2714) in *Lactobacillus plantarum*, surmised as a transmembrane protein involved in regulating polysaccharide synthesis (Brown et al., 2011; Purcell and Tamayo, 2016). The well-known probiotic strain *L. acidophilus* is one of the major species generally recognized as safe (GRAS; Martínez et al., 2012)*. L. acidophilus* is Gram-positive, produces acid through fermenting sugars into lactic acid, grows readily at rather low pH values (below 5.0), and is a probiotic microorganism that mainly inhabits the human intestines, oral cavities, and vagina (Bâati et al., 2000). As a typical probiotic, *L. acidophilus* can alleviate lactose intolerance (Kim and Gilliland, 1983), abdominal pain, and irritable bowel syndrome (Rousseaux et al., 2007) as well as modulate dendritic and T cell function (Konstantinov et al., 2008). Among the intestinal microbiota, *L. acidophilus* shows a strong autoaggregation phenotype and has been demonstrated to efficiently coaggregate with some pathogenic strains *in vitro* (Collado et al., 2008). The exopolysaccharide (EPS) produced by *L. acidophilus* possesses bioactive components with various health benefits, such as antioxidative properties and inducing cytotoxicity in two colon cancer cell lines (Deepak et al., 2016). Meanwhile, EPS also plays an important role in protecting microbes from adverse conditions, such as lysozyme osmosis as well the presence of bacteriophages, copper ions, or nisin (Looijesteijn et al., 2001).

In this study, we evaluated the possible role of c-di-GMP in regulating the probiotic properties of *L. acidophilus* for the first time. We identified the genes and operons related to the c-di-GMP signaling pathway by bioinformatic and transcriptional analyses of *L. acidophilus*. Soluble proteins were expressed and purified for subsequent evaluation. *In vivo* and *in vitro* activity assays were performed for assessing the function of c-di-GMP-related enzymes. We also confirmed a c-di-GMP-specific receptor by an *in vitro* binding test. The proteins (LA14_RS07015 and LA14_RS07020) were overexpressed *in vivo* to monitor relevant phenotypes that may be associated with c-di-GMP modulation. The regulatory function of c-di-GMP related genes in EPS formation was also evaluated in *L. acidophilus*.

## Materials and methods

### Bioinformatics

Gene identities for annotated c-di-GMP-related proteins of *L. acidophilus* La-14 were obtained from the NCBI genome (RefSeq: NC_021181.2). Conserved domain analysis was derived from the SMART (http://smart.embl-heidelberg.de/) and Pfam (Finn et al., 2016) databases. Signal peptide and transmembrane helices were predicted using SignalP 4.0 (Petersen et al., 2011) and TMHMM 2.0 (Möller et al., 2001), respectively. Soft Berry BPROM (Solovyev and Salamov, 2011) and ProOpDB (Taboada et al., 2012) were employed to predict bacterial promoters and operons, respectively. Comparative alignment and homologous proteins searching were performed using NCBI COBLAT and BLASTP, respectively (Papadopoulos and Agarwala, 2007).

### Strain construction

Putative DGC and glycosyltransferase (*gts*) genes were cloned into the pBAD-Myc-His vector carrying an ampicillin resistance gene and an L-arabinose-inducible promoter (Table 1). For measuring enzymatic activity and binding assays *in vitro*, the genes of interest were cloned into pMAL-c2, which contains a maltose-binding protein (MBP) for purification. The constitutively expressing plasmid pMG36e was used to express DGC and PDE proteins in *L*. *acidophilus* La-14. *Escherichia coli* was routinely grown in LB medium containing relevant antibiotics and under appropriate temperatures. *L*. *acidophilus* was grown in MRS medium containing relevant antibiotics at 37°C and was transformed via electroporation as described previously (Lin R. et al., 2017). Briefly, cells were cultured in MRS broth medium with 0.05% cysteine-HCl at 37°C for 48 h until optical density at 600 nm (OD_600_) reached 0.6. The culture was then diluted 1:25 in 100 mL of MRS broth with 0.5 M sucrose and 0.05% cysteine-HCl and left to grow for ~24 h until OD_600_ reached 0.8. The culture was cooled for 10 min and then cell pellets were harvested and washed twice with 0.5 M sucrose buffer, followed by an additional wash with transformation buffer (10 mM ammonium and 0.5 M sucrose; pH 6.0) and re-suspension in 400 μL transformation buffer. The recombinant plasmid was transformed into *L. acidophilus* cells by electroporation using a MicroPulser™ Electroporator (Bio-Rad, Hercules, CA, USA) at 1.5 Kv/cm. Transformed bacteria were re-suspended in MRS broth and cultured at 37°C for 1 h, followed by plating on MRS agar (1.5% w/v) containing 0.5 μg/mL erythromycin and incubation at 37°C for 48 h. Positive colonies of transformed bacteria were identified by PCR and target gene sequencing.

**Table 1:** Strains, plasmids, and primers used in this study.

**Type**	**Description**	**Reference**
**STRAIN**		
***Escherichia coli***		
Top10	Strain used for plasmid maintenance	New England Biolabs (NEB)
BL21	Strain used for overexpression of MBP-fusion proteins, Congo red staining assays, and biofilm formation assays	NEB
MG1655	Strain used for swarming and co-aggregation assays	Guangdong Microbial Culture Collection Center (GDMCC)
C43 (DE3)	Strain used for overexpression of MBP-fusion proteins	Our laboratory stock
***Lactobacillus acidophilus***		
La-14	Wild type	GDMCC
**PLASMID**		
pBAD/Myc-His-C	Vector for arabinose-inducible expression	Thermo Fisher Scientific
pBAD-*dgcA*	pBAD::*dgcA*	This work
pBAD-*gts*	pBAD::*gtsA-gtsB*	This work
pMAL-c2	Vector for IPTG-inducible expression	NEB
pMAL-*pdeA*	pMAL-c2::*pdeA*	This work
pMAL-*pdeB*	pMAL-c2::*pdeB*	This work
pMAL-EAL*_*pdeB*_*	pMAL-c2::*pde B* (EAL domain)	This work
pMAL-*ycgR*	pMAL-c2::*ycgR*	This work
pMG36e	*L. acidophilus* chromosome-integrated expression vector	Lin R. et al., 2017
pMG36e- *dgcA*	pMG36e::*dgcA*	This work
pMG36e- *pdeA*	pMG36e::*pdeA*	This work
**PRIMERS**		
pMAL-*pdeA*-F	GTCTGTCGACATGTATAAGTGGCATAATGTG	This work
pMAL-*pdeA-*R	GTCTCTGCAGTTAATAAATGTCTTCTAATTTGAGTG	This work
pMAL-*pdeB*-F	GTCTGGATCCATGGTTAAATTAATATCTATTTTAACG	This work
pMAL-*pdeB-*R	GTCTGTCGACTTATTTAATTTGTTGTGGCTTTTG	This work
pMAL-EAL*_*pdeB*_*-F	GTCTGGATCCCAAAAAACAGGCATAGATGAAG	This work
pMAL-EAL*_*pdeB*_*-R	TCTGTCGACCAAAAAACAGGCATAGATGAAG	This work
pMAL-*ycgR*-F	GTCTGGATCCGTGAGTCATTACCATGAGCAG	This work
pMAL-*ycgR*-R	GTCTGTCGACTCAGTCGCGCACTTTGTCCG	This work
pBAD-*dgcA*-F	GTCTCTCGAGTGTGTTTTTTCAAGTCTTAAGC	This work
pBAD-*dgcA-*R	GTCTCTGCAGTTAACCAATTAGGATTTTTGC	This work
pBAD-*gts*-F	GTCTCTCGAGGTGAACATAGATAAAGATGTCGAAG	This work
pBAD-*gts*-R	GTCTCTGCAGTTAATCTACCTTCCGCTTAGGA	This work
pMG36e- *dgcA*-F	GTCTTCTAGAGGTGTTTTTTCAAGTCTTAAGCTC	This work
pMG36e- *dgcA*-R	GTCTAAGCTTTTAACCAATTAGGATTTTTGCTCG	This work
pMG36e- *pdeA*-F	GTCTTCTAGAGATGTATAAGTGGCATAATGTGTTTC	This work
pMG36e- *pdeA*-R	GTCTAAGCTTTTAATAAATGTCTTCTAATTTGAGTGC	This work

### Transcriptional analysis

To characterize operon regulation of the *dgcA, pdeA, pdeB, gtsA*, and *gtsB* genes, total RNA was extracted and purified. Briefly, an overnight culture of *L. acidophil*us La-14 was added into MRS medium and incubated until the late exponential phase. The cells were collected and treated with lysozyme and RNA was extracted using RNAiso reagent (Takara, Shiga, Japan). After treatment with DNA Eraser, the RNA was reverse transcribed into cDNA according to the PrimeScript RT Master Mix Kit (Takara) protocol.

### Swarming and congo red dye binding assays

Congo red binding assays were used to determine bacterial EPS production. LB (*E. coli*) or MRS (*L. acidophilus*) agar plates containing 50–80 μg/mL Congo red was treated at 30°C for 48 or 72 h. For swarming assays, LB plates were made with 0.5% agar supplemented with 0.5% L-arabinose (Harshey and Matsuyama, 1994; Paul et al., 2010). Overnight cultured cells were used to inoculate the plates and then incubated at 37°C for 24 h.

### Protein overexpression and purification

During MBP-PdeA, MBP-EAL_pdeB_ and MBP-YcgR fusion protein expression, IPTG (final concentration, 0.6 mM) was added to exponentially growing *E. coli* BL21 for a 3-h induction at 37°C. For MBP-PdeB fusion protein expression, IPTG (final concentration, 0.3 mM) was added to exponentially growing *E. coli* C43 for a 12-h induction at 30°C. After induction, cell pellets were harvested by centrifugation at 6,000 × *g* for 10 min. Cell pellets were resuspended in lysis buffer containing 150 mM NaCl, 1 mM EDTA, 5 mM MgCl_2_, 20 mM Tris-HCl (pH 7.4), 17 μg/mL PMSF, and protease inhibitor cocktail (Roche, Basel, Switzerland). After sonication and centrifugation, the clarified lysates were loaded onto a pre-equilibrated amylose column (NEB, Ipswich, MA, USA) which was subsequently washed with 12 column volumes of column buffer (150 mM NaCl, 1 mM EDTA, and 20 mM Tris-HCl, pH 7.4). MBP-fusion proteins were eluted with column buffer containing 10 mM maltose that was subsequently exchanged with PDE activity assay buffer or c-di-GMP binding assay buffer using Amicon Ultra-15 mL Centrifugal Filter Units (Merck Millipore, Burlington, MA, USA). Purified proteins were detected by 12% sodium dodecyl sulfate-polyacrylamide gel electrophoresis (SDS-PAGE) and protein concentration was measured by the BCA protein assay (Pierce, Rockford, IL, USA).

### PDE assays

PDE assays were performed as previously described (Schmidt et al., 2005). Briefly, the PDE assay components were incubated with 10 μM enzyme (MBP-PdeA or MBP-EAL_pdeB_) for 1 h at 37°C in buffer containing 50 mM Tris-HCl (pH 9.35), 5 mM MgCl_2_, 50 mM NaCl, 0.5 mM EDTA, and 100 μM c-di-GMP (Biolog, Bremen, Germany). To monitor the hydrolysis rates of c-di-GMP, the reactions were stopped by adding CaCl_2_ (final concentration, 10 mM) at various time points and then samples were boiled for 5 min and centrifuged. The supernatant was filtered through a 0.22 μm filter and analyzed by reversed-phase high performance liquid chromatography (HPLC; Waters, Milford, MA, USA). Reactants (15 μL) were injected into a TC-C18 column (15 × 4.6 cm; Agilent, Santa Clara, CA, USA) and separated by reversed-phase HPLC with a buffer system based on the gradient program described previously (Ryjenkov et al., 2005).

### c-di-GMP binding assays

Differential radial capillary action of ligand assay (DRaCALA) was performed as described previously (Fang et al., 2014) with some modifications. Briefly, MBP-fusion protein in binding buffer (300 mM NaCl, 1 mM EDTA, 10% glycerol, and 50 mM Tris-HCl, pH 7.5) was mixed with 0.5 μM 2′-fluo-aminohexylcarbamoyl-c-di-GMP (Fluo-c-di-GMP; Biolog) and incubated for 20 min at room temperature. Fluo-c-di-GMP was competed away with cold nucleotides in different concentrations. Then, 2 μL of the mixture was spotted on nitrocellulose membranes (Merck Millipore) in triplicate. The Typhoon FLA 9000 scanner (excitation wavelength, 473 nm; GE Healthcare, Chicago, IL, USA) was used to detect membrane fluorescence. The dissociation constant of specific protein-ligand interactions was measured by altering the protein concentration.

Equilibrium dialysis experiments were performed as previously described (Ryjenkov et al., 2006). MBP-EAL_pdeB_ (16 μM) was placed into one chamber of the Dispo Equilibrium DIALYZER (10 kDa cut off; Harvard Apparatus, Holliston, MA, USA) with binding buffer. C-di-GMP (1–50 μM) in an equivalent volume (70 μL) was placed in the other chamber. The dialyzers were slowly agitated for 24 h at room temperature to reach equilibrium. Samples from each chamber were boiled for 5 min and centrifuged. The supernatants were then filtered through a 0.22 μm micro filter. For quantification, 50 μM of GDP (final concentration) was added to each sample. Reactants (15 μL) were injected into a TC-C18 column (15 × 4.6 cm; Agilent) and separated by reversed-phase HPLC with a buffer system based on the gradient program described previously (Ryjenkov et al., 2005).

### Biofilm and EPS formation assays

The ability of bacteria in forming stable biofilms was assessed using cells growing in 96-well plates according to a previous method (O'Toole and Kolter, 1998) with some modifications. For *E. coli*, different concentrations of L-arabinose were added to the exponentially growing cultures (OD_600_ = 0.6–0.7) and then 200-μL aliquots of each culture were used to inoculate each of four wells. Plates were incubated at 30°C for 24 h. For biofilm quantification, the media were discarded from microtiter plates to remove unbound cells and then the plates were gently washed twice by TBS. After air-drying, the adherent bacteria were stained with 100 μL 0.1% crystal violet for 15 min at room temperature and then the plates were gently washed twice. The bound dye was extracted from the stained cells by adding 200 μL of an ethanol/acetone (8:2) mixture. Biofilm formation was then quantified by measuring OD_600_.

EPS formation was evaluated with Congo red dye binding assays and confocal laser scanning fluorescence microscopy as described previously (Wu et al., 2016). *L. acidophilus* La-14 and its derivatives were grown in MRS broth with 0.5 μg/mL erythromycin for 24 h and the cultures were harvested and diluted 1:100 with MRS medium, after which 5 mL of diluted culture was added to 6-well plates with coverslips placed at the bottom of each well. After incubation for 120 h in 5% CO_2_ at 37°C, the coverslips were gently washed twice with sterile Tris-buffered saline (TBS) to remove unbound bacteria and then stained with calcofluor-white (Sigma-Aldrich, St. Louis, MI, USA) for 15 min at room temperature in the dark to stain the EPS. The coverslips were then gently washed two times with sterile TBS and observed with a Nikon A1 confocal laser microscope (Nikon, Tokyo, Japan) using the 351-nm line. The stained EPS then appeared blue during confocal fluorescence microscopy analysis. At least five independent fields were collected at 60 × magnification per experiment and three independent experiments were performed. Image J software (version 1.43; NIH) was used to calculate the area covered by the germs.

### Co-aggregation assays

Co-aggregation assays were performed as previously described (Collado et al., 2008; Johnson and Klaenhammer, 2016) with some modifications. Bacterial suspensions for co-aggregation were prepared following the autoaggregation assay protocol. Then, the same volumes of cell suspensions (1 mL) of different probiotic and pathogenic strains were mixed together in pairs and vortexed for 10 s and incubated at room temperature without agitation. OD_600_ of the suspensions were measured during a 5-h incubation period. The percentage of co-aggregation was calculated using $\left[\frac{\text{(Apat + Aprobio)}}{2}-\text{Amix}\right]/\left[\frac{\text{(Apat + Aprobio)}}{2}\right]\times 100$, where Apat and Aprobio represent the OD_600_ of pathogenic and probiotic bacterial suspensions, respectively, and Amix represents the mixture OD_600_ at different time points.

## Results

### Analysis of genes related to the c-di-GMP signaling pathway in *L. acidophilus*

**Figure 1:**
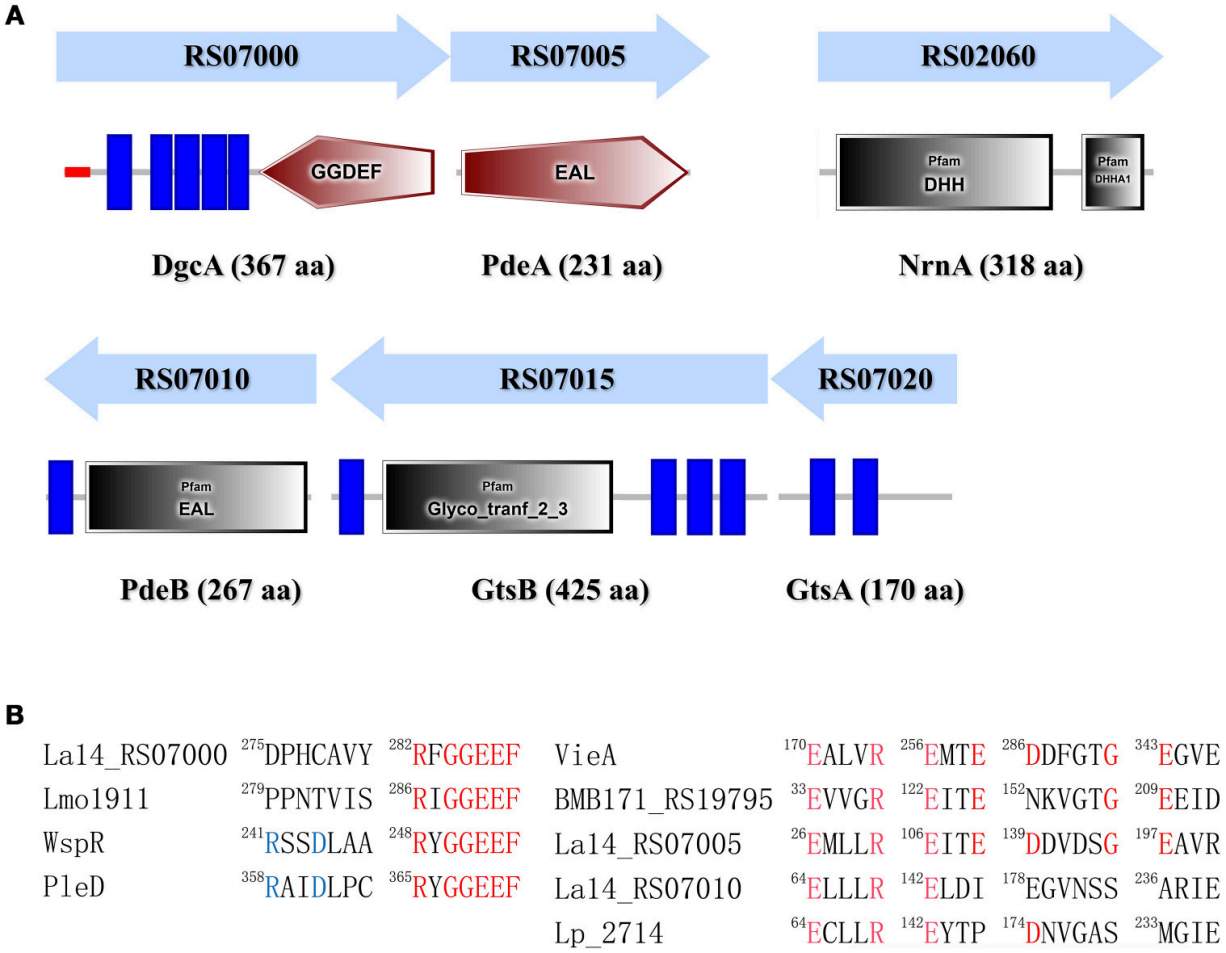
Identification of proteins involved in the c-di-GMP signaling pathway in *Lactobacillus acidophilus* La-14. **(A)** Domain symbols are derived from the SMART and Pfam database. In the upper panel, the domain structures are shown under the predicted operon arrangements with gene numbers. The putative name and number of amino acids in each protein are listed at the bottom. Predicted signal peptide and transmembrane regions are shown in red stripes and blue rectangles, respectively. **(B)** Amino acid sequence alignment of the conserved active site residues for the GGDEF domain (left) and the EAL domain (right). Residues known to be conserved and catalytically important are shown in red. The RXXD motif of the I-site for c-di-GMP binding is shown in blue. Experimentally characterized GGDEF domains are from *L. monocytogenes* (Lmo1911) (Chen et al., 2014), *Pseudomonas aeruginosa* (WspR, De et al., 2009), and *Caulobacter crescentus* (PleD, Paul et al., 2004). Experimentally proven EAL domain sequences are from *Vibrio cholerae* (VieA) (Tamayo et al., 2005), *Bacillus thuringiensis* (BMB171_RS19795, Fu et al., 2018), and *L. plantarum* (LP_2714, Brown et al., 2011).

C-di-GMP is synthesized by DGC from two GTP molecules and is hydrolyzed by PDE to pGpG. DGC family proteins contain a conserved Gly-Gly-Asp-Glu-Phe (GGDEF) sequence motif, whereas PDE family proteins contain a conserved EAL or HD-GYP motif. The *L. acidophilus* La-14 genome (NCBI reference sequence: NC_021181.2) contains a gene (LA14_RS07000, *dgcA*) encoding the GGDEF domain and two genes (LA14_RS07005, *pdeA*; LA14_RS07010, *pdeB*) encoding the EAL domain (Figure 1A); these genes may be involved in the metabolic cycle of c-di-GMP. The EAL-only proteins (PdeA and PdeB) can serve as either active PDEs (class I) or inactive enzymes (class III; El Mouali et al., 2017).

*L. acidophilus* La-14 has only one GGDEF domain-containing protein (DgcA; NCBI reference sequence, WP_011254455.1) associated with DGC activity. The N-terminal domain of the predicted DGC protein contains one signal peptide and five transmembrane helices that may sense external signals to regulate c-di-GMP synthesis (Figure 1A). Amino acid sequence alignment (Figure 1B) showed that La14_RS07000 possesses a conserved active site (RxGGDEF) but lacks an inhibitory site (RxxD), similar to *L. monocytogenes* Lmo1911 (Chen et al., 2014).

The EAL domain protein (Figure 1), La14_RS07005 (PdeA; WP_011254456.1), contains only one EAL domain with conserved residues for c-di-GMP hydrolysis (Tchigvintsev et al., 2010). Bioinformatic analysis predicted that it also lacks the conserved loop 6 [DFG(A/S/T)(G/A)(Y/F)(S/A/T)(S/A/G/V/T)] and adjacent domain that can potentially promote dimerization for enhancing enzymatic activity (Rao et al., 2009). La-14 shared extensive similarity with the NCFM strain during alignment of *L. acidophilus* genomes (Stahl and Barrangou, 2013). According to ProOpDB, *dgcA* and *pdeA* were predicted to belong to the same operon in strain NFCM, whereas we found the opposite prediction in strain La-14. Subsequent biochemical analyses were needed to clarify this contradiction (see section Operon Transcriptional Analysis). The amino acid sequence of another EAL domain protein, La14_RS07010 (PdeB; WP_003548090.1; Figure 1), contains two fractions, a membrane targeting signal sequence and an EAL domain without the residues required for catalysis. Although PdeB appears to lack hydrolysis ability, it retains the c-di-GMP binding site and the conserved EXLXR motif, suggesting that it acts as a receptor protein as previously described (Minasov et al., 2009; Chou and Galperin, 2016). From the c-di-GMP census [http://ncbi.nlm.nih.gov/Complete_Genomes/c-di-GMP.html], there is no other predictable c-di-GMP receptor except for PdeB from the sequence analysis of *L. acidophilus* NCFM. Thus, the neighboring genes of *pdeB*—*La14_RS07015* and *La14_RS07020*—emerged as the main genes of interest in our study.

The GtsB protein (WP_011254457.1), encoded by the *La14_RS07015* gene nearby *pdeB*, was described as a glycosyltransferase that functions in the synthesis of cellulose, which is similar to BcsA and PgaC function in *Rhodobacter sphaeroides* and *E.coli*, respectively (Steiner et al., 2013; Morgan et al., 2014). Overall, BcsA and GtsB shared 25% amino acid identity and 37% sequence similarity and both belong to glycosyltransferase family 2. GtsB contains an N-terminal and three C-terminal transmembrane domains as well as a predicted cytoplasmic glyco_tranf_2_3 domain (Figure 1A). GtsA (WP_003548094.1), encoded by *La14_RS07020* upstream of the *pdeB* and *gtsB* genes, was predicted to be a transmembrane protein without any conserved domains. Similar to PgaC-PgaD complex, the membrane-anchored GtsA subunit, together with the GtsB, may form a glycosyltransferase complex (Steiner et al., 2013).

In *Pseudomonas aeruginosa*, the intermediate molecule pGpG, produced by EAL domains were confirmed to be eventually hydrolyzed to GMP by oligoribonuclease (Cohen et al., 2015). Firmicutes lacks oligoribonuclease but have its homologs protein family nanoRNases (Nrn; clusters of orthologous group: COG0618) instead (Orr et al., 2015). In NCBI protein database, we found an oligoribonuclease functional homologs NrnA (La14_RS02060, WP_011254146.1) which may be responsible for degradation of pGpG in *L. acidophilus* La-14 (Figure 1A). Besides, according to known c-di-GMP receptors, BlastP was used to search the homologous proteins in La-14. Several putative c-di-GMP receptors were listed in Table 2, but their binding capacity should be confirmed by the biochemical analyses.

**Table 2:** Putative c-di-GMP receptors in *L. acidophilus* La-14.

**Known c-di-GMP receptors, organism**	**UniProt entry**	**References**	**Homologous protein in La-14**
Bcam1349, *Burkholderia cenocepacia*	B4EIC5	Fazli et al., 2011	NA
BdcA, *E. coli*	PF00106	Ma et al., 2011	LA14_RS05540
BcsE, *E. coli*	P37657.1	Fang et al., 2014	NA
BrlR, *P. aeruginosa*	Q9HUT5	Chambers et al., 2014	LA14_RS09635 LA14_RS05180
CLP, *Xanthomonas campestris*	P22260	Chin et al., 2010	NA
PgaC, *E. coli*	P75905	Steiner et al., 2013	LA14_RS07015 LA14_RS00530
PgaD, *E. coli*	P69432	Steiner et al., 2013	NA
VpsR, *V. cholerae*	Q9KU59	Srivastava et al., 2011	NA
PA4608, *P. aeruginosa*	1YWU_A	Ramelot et al., 2007	NA
VpsT, *V. cholerae*	Q9KKZ8	An et al., 2014	NA

### Operon transcriptional analysis

**Figure 2:**
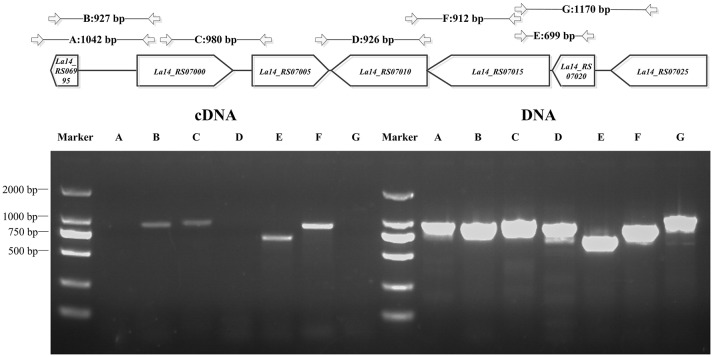
PCR for *L. acidophilus* transcriptional analysis. Upper panel: genes of interest (in the box) on the chromosome and the length of amplified PCR products (in bp) with specific primer pairs (Table S1) that span the sequences, *dgcA* to its −831 or −638 bp positions, *dgcA* to *pdeA, pdeA* to *pdeB, gtsA* to *gtsB, gtsB* to *pdeB*, and *La14_7025* to *gtsA*. Lower panel: 1% agarose gel PCR analysis of *dgcA, pdeA, pdeB, gts*, and adjacent genes with specific primer pairs that were used to amplify both gDNA (right half) and cDNA (left half) from *L. acidophilus*. The letters A–G correspond to the amplified products of gene sequences indicated in the upper panel. The results indicate that the amplified A, D, and G PCR products are non-consecutive, while B, C, E, and F are consecutive in cDNA. Lanes: M, 2,000 bp DNA Ladder; cDNA, La-14 complementary DNA; DNA, La-14 genomic DNA.

Through bioinformatics prediction, a promoter region at position −264 or −653 upstream of *dgcA* was found. Amplified product A (*dgcA* to position −831) contained both predicted promoter regions, while amplified product B (*dgcA* to position −638) only contained the promoter region at −264. Based on the principle that promoter sequences can't be transcribed, the corresponding size of B appeared while the A fragment did not (Figure 2), suggesting that the promoter sequence of *dgcA* is at position −653. Amplified products in the C (*dgcA* to *pdeA*), E (*gtsA* to *gtsB*), and F (*gtsB* to *pdeB*) regions indicate that *dgcA* and *pdeA* form an operon, while *pdeB, gtsB*, and *gtsA* form another operon named *gts* on the *L. acidophilus* chromosome. Therefore, the results suggest that *dgcA* and *pdeA* are under the control of a single promoter in an operon and are involved in c-di-GMP cycling.

### DGC activity assays *in vivo*

**Figure 3:**
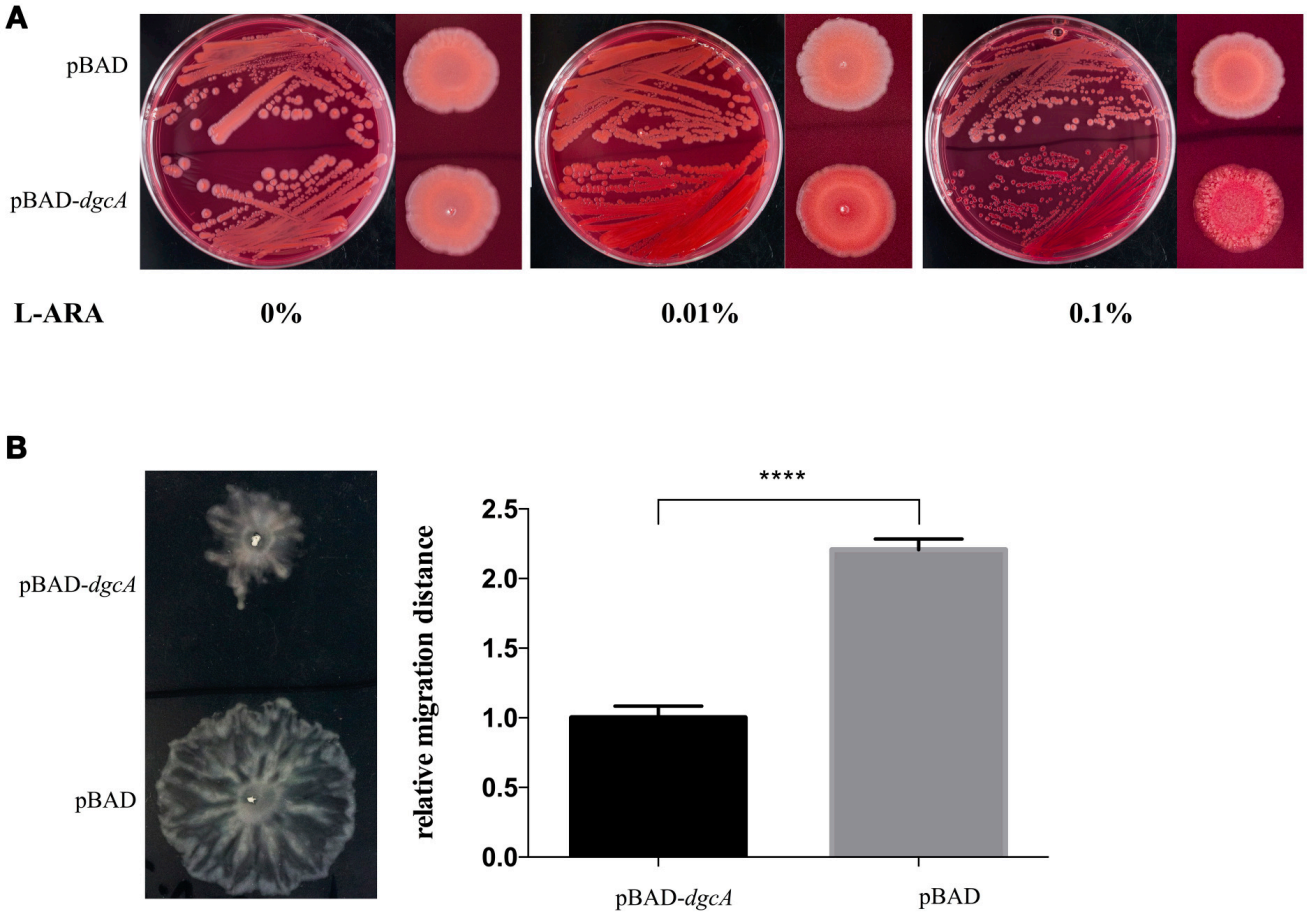
Congo red staining and swarming motility assays. **(A)** Congo red staining of the EPS-producing strain BL21 caused by *L. acidophilus* DgcA, indicative of its DGC activity. DgcA was expressed with the plasmid pBAD-Myc-His. LB agar contained 0–0.1% L-arabinose. **(B)** Inhibition of motility in swarming plates (0.5%) of strain MG1655 by DgcA supports its DGC activity. LB agar contained 0.5% arabinose. Average results from three independent tests of the swarm zones. ^****^significant difference (*p* < 0.0001). GraphPad Prism 6.1 was used to perform Student's *t*-tests.

The DGC activity of *L. acidophilus* DgcA was analyzed by Congo red staining and swarming motility assays on Congo red plates and 0.6% agar plates, respectively. The binding of Congo red was associated with the production of EPS or curli fimbriae (Olsén et al., 1989). When concentrations of the inducer L-arabinose increased, colonies expressing DgcA were red-stained, dry, and rough compared with the empty vector-containing negative control (Figure 3A). In swarming motility assays for assessing another c-di-GMP-dependent phenotype, the motility of *E. coli* MG1655 containing the pBAD-*dgcA* plasmid was highly inhibited compared with the control group (Figure 3B). Both assays suggested that colonies expressing DgcA contained a higher content of c-di-GMP.

### PDE activity assays *in vitro*

**Figure 4:**
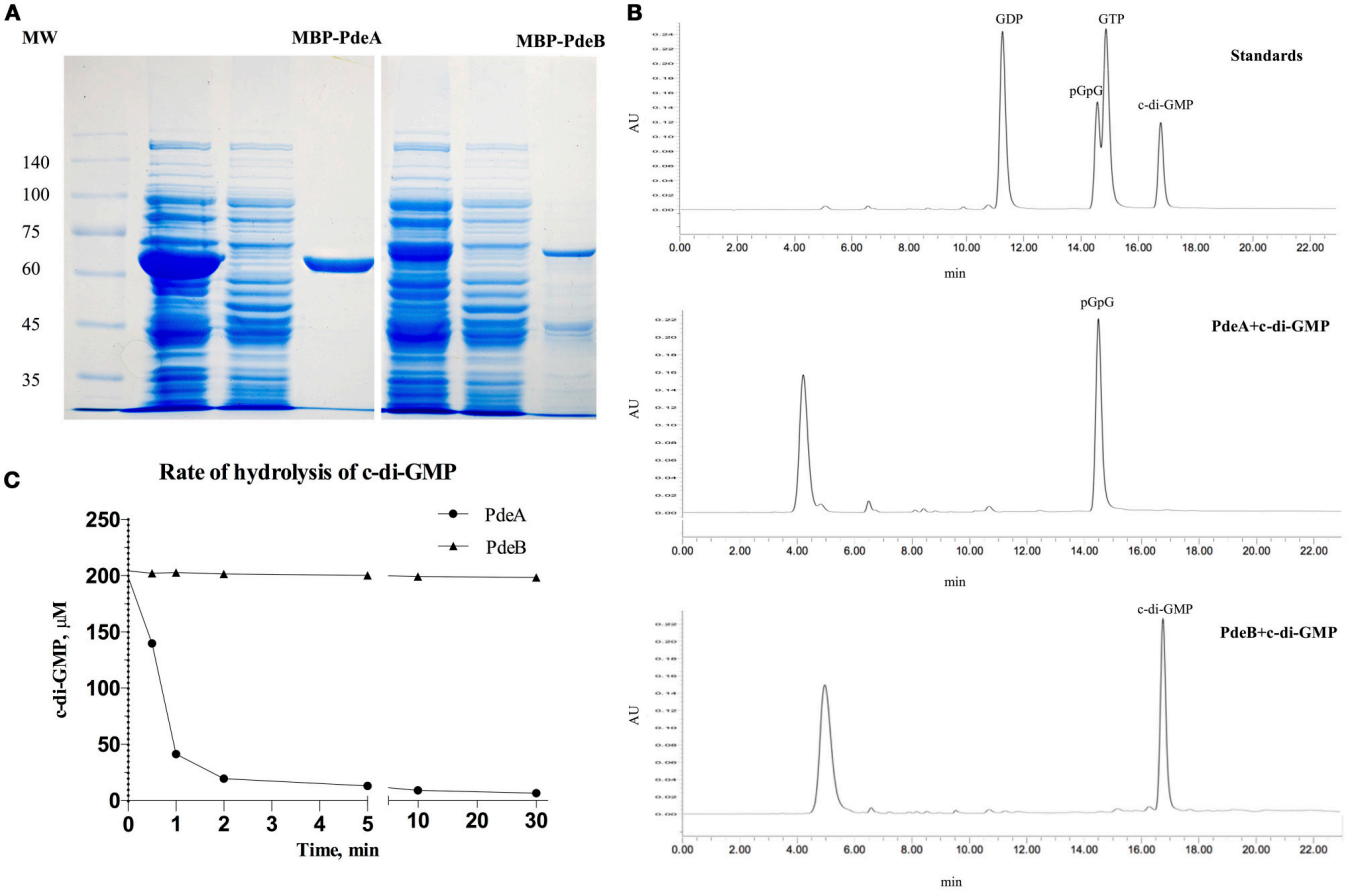
Phosphodiesterase (PDE) activity and HPLC assays. **(A)** 12% SDS-PAGE of *L. acidophilus* PdeA (MBP-PdeA) and PdeB (MBP-PdeB) affinity purification to be used in PDE assays. **(B)** HPLC chromatograms showing the standards and products of PDE assays. **(C)** PDE activities of MBP-PdeA and MBP-PdeB were monitored based on the hydrolysis rate of c-di-GMP measured by HPLC.

To directly measure c-di-GMP PDE activity *in vitro*, PdeA and PdeB were expressed and purified to determine whether they can hydrolyze c-di-GMP. PdeA and PdeB were purified as MBP-fusion proteins (Figure 4A). The PdeA enzymatic reaction product corresponded to the retention time of the pGpG [5′-phosphoguanylyl-(3′ → 5′)-guanosine] standard, indicating that PdeA was able to hydrolyze c-di-GMP to pGpG. However, the PdeB enzymatic reaction product corresponded to the retention time of the c-di-GMP standard, indicating that PdeB does not possess PDE activity *in vitro* (Figures 4B,C).

### PdeB protein is a c-di-GMP receptor

**Figure 5:**
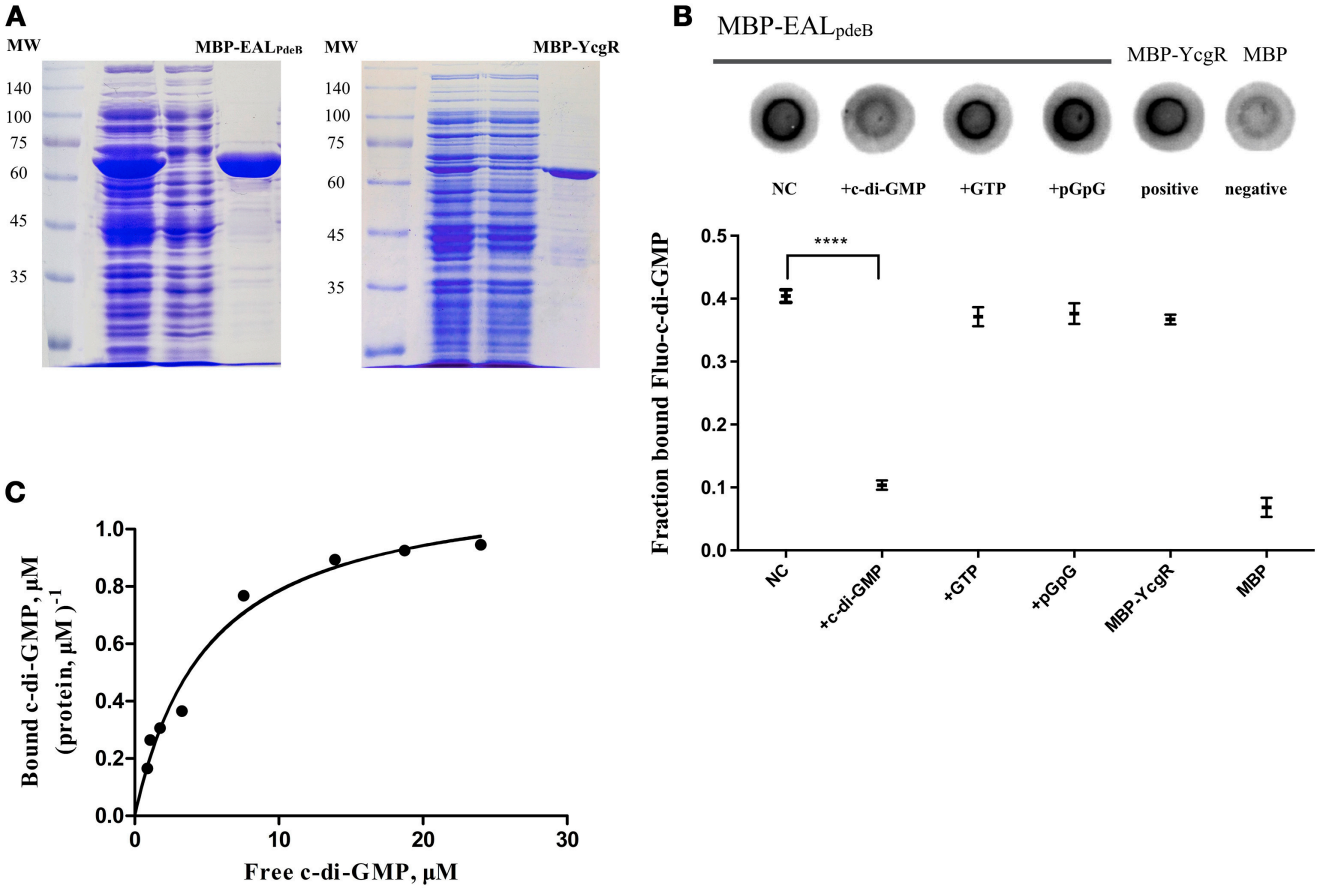
DRaCALA and equilibrium dialysis experiments for the c-di-GMP-specific receptor. **(A)** SDS-PAGE of MBP-EAL_pdeB_ and MBP-YcgR overexpressed in BL21 and purified with amylose resin. The EAL domain of PdeB (residues 32–267) with an ELLLR motif was fused with MBP and used in c-di-GMP binding assays. **(B)** Binding of Fluo-c-di-GMP to PdeB (MBP-EAL_pdeB_) in DRaCALA. Upper panel: Fluo-c-di-GMP in the presence of increasing concentrations of unlabeled c-di-GMP as a competitor. Lower panel: graph of F_B_ for each sample with averages indicated by a horizontal bar of three independent experiments. ^****^Significant difference (*p* < 0.0001). GraphPad Prism 6.1 was used to perform Student's *t*-tests. **(C)** Equilibrium binding between c-di-GMP and MBP-EAL_pdeB_. The chart represents the ratio of bound c-di-GMP per protein unit in the dialysis chamber vs. the concentration of free c-di-GMP in another chamber.

To demonstrate that the PdeB protein acts as a c-di-GMP-specific receptor, we overexpressed its EAL domain (MBP-EAL_PdeB_) containing the ELLLR substrate binding site as an MPB-fusion protein (Figure 5A) and tested its ability to bind c-di-GMP through DRaCALA and equilibrium dialysis. According to the results of the competitive binding assay, excessive unlabeled c-di-GMP competed for Fluo-c-di-GMP and MBP-EAL_PdeB_ binding effectively (*P* < 0.001; Figure 5B), whereas GTP and pGpG did not, indicating that PdeB can bind c-di-GMP specifically. EAL_PdeB_ bound c-di-GMP with a *K*_*d*_ of 4.871 ± 0.89 μM and a B_max_ of 1.158 ± 0.07 μM c-di-GMP (μM protein) ^−1^ (Figure 5C). *K*_*d*_ value was in the range of 0.1–13 μM, which was consistent with the *K*_*d*_ ranges of other EAL domain (EXLXR motif)-based proteins (FimX or LapD; Chou and Galperin, 2016).

### Overexpression of GtsA and GtsB increases EPS synthesis in *E. coli*

**Figure 6:**
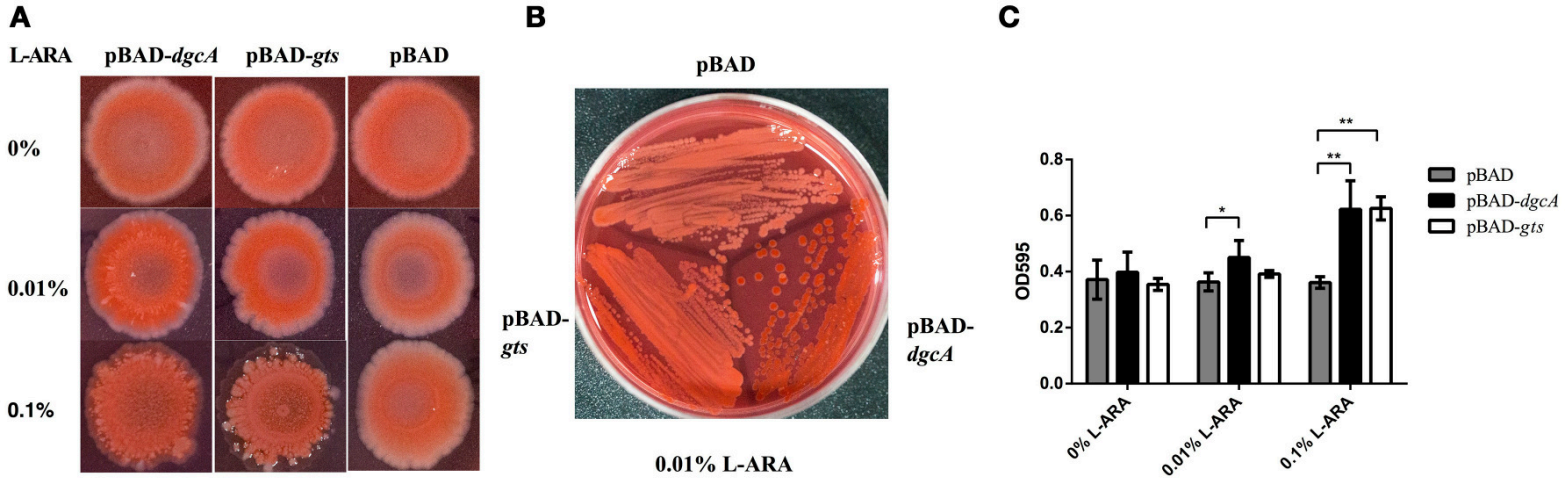
Congo red staining and biofilm formation assays. **(A,B)** Congo red staining of the EPS-producing strain BL21 was caused by *L. acidophilus* DgcA or Gts. Proteins were expressed by cloning the corresponding genes into the pBAD-Myc-His vector. LB agar contained 0–0.1% L-arabinose. **(C)** Biofilm formation of *E. coli* BL21 transformed with different recombinant plasmids in 96-well plates with 0–0.1% L-arabinose. Bars denote mean values of the data from three biological replicates. ^*^significant difference (*p* < 0.05); ^**^significant difference (*p* < 0.01). GraphPad Prism 6.1 was used to perform Student's *t*-tests.

Cellulose and poly-N-acetylglucosamine increase biofilm formation of *E. coli* on abiotic surfaces (Wang et al., 2004). To analyze the function of the *gts* operon, we cloned its coding sequence of *gtsA* and *gtsB* into the pBAD-Myc-His vector, followed by *E. coli* (BL21) transformation. Utilizing heterologous expression allowed us to assess the effects of the protein of interest without additionally impacting protein-protein interactions. On Congo red plates, compared with control, the expression of pBAD-*gts* resulted in red color colonies but with a lesser extent than pBAD-*dgcA* (Figures 6A,B). Moreover, we performed a crystal violet staining assay to examine biofilm formation. Under induction, an increase in biofilm formation was observed in the colonies expressing pBAD-*dgcA* and pBAD-*gts* (Figure 6C). The pBAD-*gts* group exhibited a somewhat similar phenotype to the pBAD-*dgcA* group, which may be due to the lack of PdeB c-di-GMP receptor activation. These results suggest that *gts* is associated with the formation of bacterial EPS.

### Intracellular DgcA and PdeA levels regulate bacterial form and EPS formation in *L. acidophilus*

**Figure 7:**
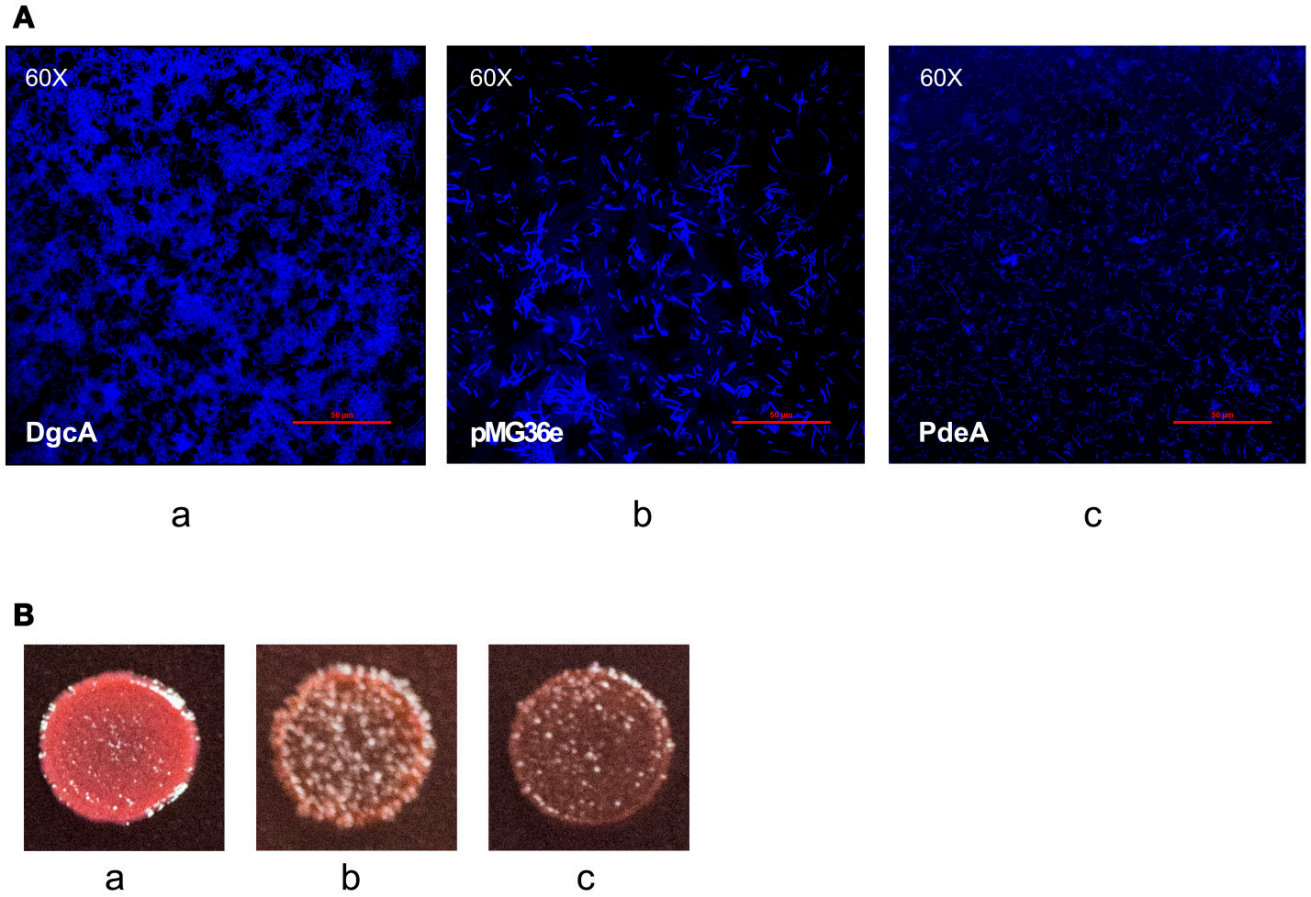
EPS formation analysis with confocal microscopy and Congo red staining. **(A)** Confocal microscopy for examining (a) La-14::*dgcA*; (b) La-14::pMG36e; (c) La-14::*pdeA*. The blue color indicates EPS formation. **(B)** Congo red staining of the La-14 strain is indicative of EPS production. (a) La-14::*dgcA*; (b) La-14::pMG36e; (c) La-14::*pdeA*.

After analyzing the functional components of c-di-GMP signaling in *L. acidophilus*, we determined the phenotypes associated with increased intracellular DgcA or PdeA levels. We overexpressed DgcA or PdeA in *L. acidophilus* La-14 and analyzed their roles in EPS formation. The covered area on the coverslip surface by La-14 and its recombinant strains was shown in Table 3. Compared with the vector control, the strain expressing DgcA adhered more biomass to the coverslip, formed a smaller size of cell and more compact structures (Figure 7Aa), whereas the strain expressing PdeA grew in short rod-shaped chains, and stained in lighter blue (Figure 7Ac). Among these photos, the difference of EPS production level was not obvious. Then we performed a Congo red assay to detect EPS and found that the strain expressing DgcA exhibited redder colonies compared with other strains (Figure 7B). This result suggest that DgcA may promote the formation of EPS in *L. acidophilus*.

**Table 3:** Cover area on the surface by the recombinant strains of La-14.

	**La-14::*dgcA***	**La-14::pMG36e**	**La-14::*pdeA***
Cover area (%)	48.47 ± 7.34^**^	14.93 ± 4.45	11.76 ± 2.01

*Statistical results from three independent tests of the cover area*.

** *significant difference vs. La-14::pMG36e (p < 0.01). GraphPad Prism 6.1 was used to perform Student's t-tests*.

### DgcA/PdeA-induced *L. acidophilus* EPS promotes co-aggregation with *E. coli*

**Figure 8:**
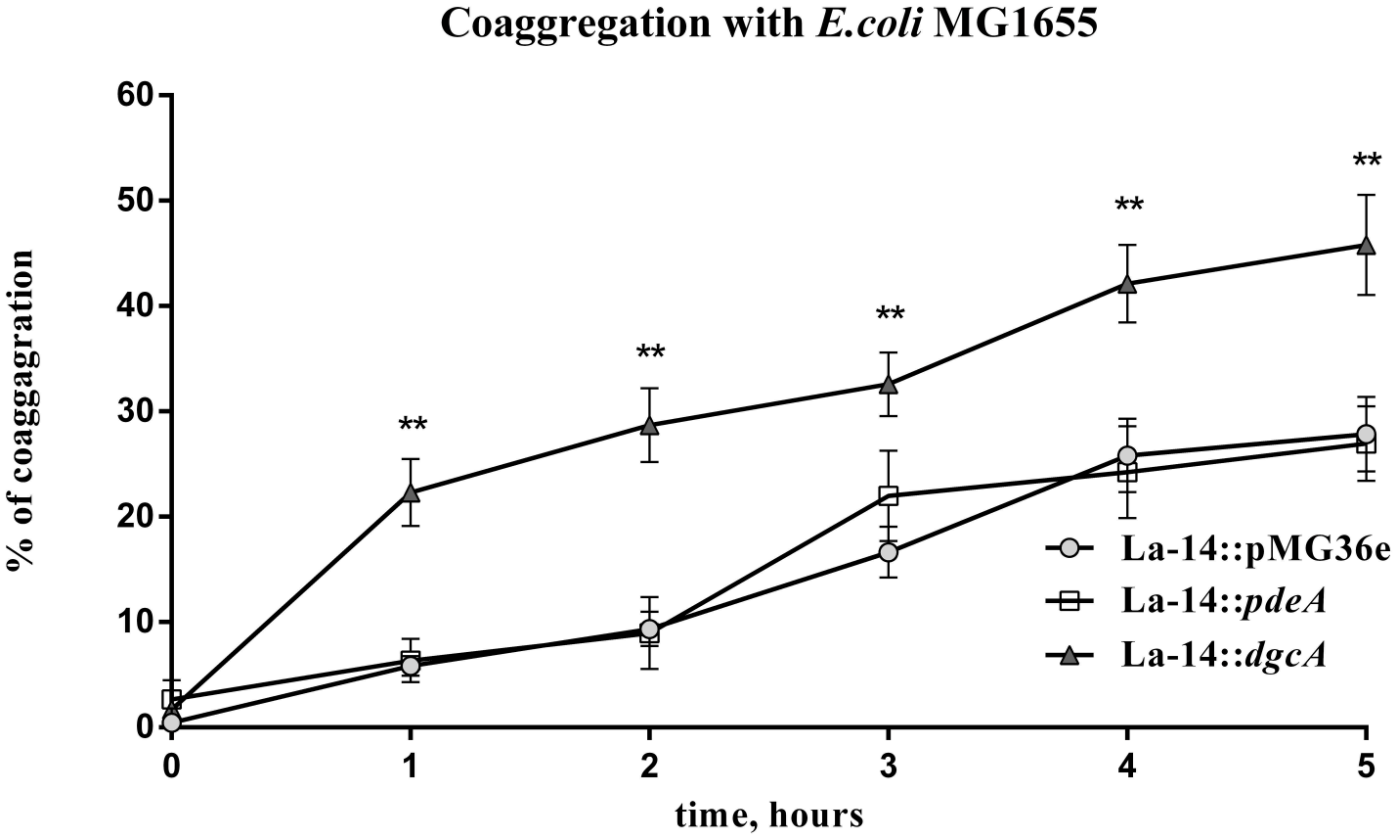
*L. acidophilus* co-aggregation assay with *E. coli* MG1655. Co-aggregation abilities of recombinant La-14 with potential pathogen *E. coli* MG1655 were measured in 96-well polystyrene plates. The triangle represents La-14::*dgcA*; circle, La-14::pMG36e; square, La-14::*pdeA*. Data represent the average results from two independent experiments of each strain grown in three wells. ^**^significant difference (*p* < 0.01) between La-14::*dgcA* and control group (La-14::pMG36e). GraphPad Prism 6.1 was used to perform Student's *t*-tests.

In the presence of other bacteria or fungi, lactobacilli strains usually exhibit strong co-aggregation phenotypes, which is a characteristic of probiotics (Collado et al., 2008; Chew et al., 2015). To test the role of DgcA/PdeA-induced EPS formation in *L. acidophilus*, the co-aggregation of this strain compared with *E. coli* was evaluated. The settling rate was determined within 5 h. During the first hour, the three strains showed similar autogenesis rates. From 1 to 5 h, the co-aggregation rate of La-14::*dgcA* was significantly faster (*P* < 0.01) than those of La-14::pMG36e and La-14::*pdeA* (Figure 8). However, there was no significant difference between La-14::pMG36e and La-14::*pdeA* throughout the assay.

## Discussion

Bacteria adapt to environmental stresses through changes in EPS and proteinaceous appendages. Such adaptations are regulated in various bacteria by proteins with GGDEF and EAL domains, which involve the second messenger c-di-GMP. The GGDEF domain acts as a nucleotide cyclase for c-di-GMP synthesis, whereas the EAL domain acts as a phosphodiesterase for c-di-GMP degradation. As *L. acidophilus* is a probiotic and a significant bacterium present in the digestive tract, it is important to investigate the probiotic properties of probiotics. In the present study, we aimed to address whether there are c-di-GMP regulator-related genes in the *L. acidophilus* genome, how the genes are organized into a cluster to form the operons, and the roles these genes play in conducting their biological functions.

First, we collected La-14 genomic information through the NCBI (GenBank) website. Subsequently, bioinformatic analysis for c-di-GMP related genes and proteins were performed. In combination with our experimental data, we confirmed the presence of one Dgc gene (*dgcA*), two copies of Pde (*pdeA* and *pdeB*), one copy of nanoRNases gene (*nrnA*), and one copy of each Gts subunit gene, *gtsA* (subunit A) and *gtsB* (subunit B), on the *L. acidophilus* chromosome (Figure 1A). Then.we confirmed that *dgcA* and *pdeA* belong to one operon while *pdeB, gtsA*, and *gtsB* belong to another.

The conformation of GGDEF and EAL domains affects the catalytic function of DGC and PDE enzymes, respectively (Chan et al., 2004). In our experiment, PdeA exhibited PDE activities *in vitro* and DgcA induced two c-di-GMP-dependent phenotypes (low motility and high production of curli fimbriae) in *E. coli* by heterologously expressed *in vivo*. However, even though PdeB has no enzymatic activity, it can bind to c-di-GMP as a receptor. Moreover, we found that the GGDEF and EAL motifs of DgcA and PdeA, respectively, were homologous to the conserved motifs of other bacterial strains in which the two enzymes retain their catalytic activities (Figure 1B). Similarly, the amino acid sequences of DgcA and PdeA in La-14 also showed higher sequence homology with other members of the *Lactobacillus* family such as *L. amylovorus, L. kalixensis*, and *L. reuteri* (Figures S1A,B). This suggests that the c-di-GMP signaling pathway seem to be involved in *Lactobacillus* strain behavior regulation throughout evolution. However, the EAL motif of PdeB showed less homology with other known PDEs (Figure 1B). We believe that the structural differences in PdeB resulted in its lack of c-di-GMP hydrolase activity. In the EAL domain, the conserved motif DDFG(T/A)GYSS plays an important role in positioning Mg^2+^ for catalytic activity (Römling, 2009); however, there is no such motif in PdeB and instead, the EGVNSSARIE motif is present (Figure 1B). In fact, several EAL domain-containing PDEs with variations in some of these conserved residues lack PDE activity but retain a regulatory role. Furthermore, the GGDEF domain in DgcA lacks the conserved RxxD motif that is used as an inhibitory site for receiving feedback regulation by c-di-GMP in other bacterial strains (Figure 1B). DgcA expressed without the N-terminal hydrophobic sequence exhibited no activity *in vitro* (data not shown) after we observed no DgcA activity when the full-length *dcgA* gene was transformed in bacterial cells. This may be due to the truncation of its N-terminal transmembrane domain, as this domain is disadvantageous for expression of soluble proteins. In addition, lack of the transmembrane domain, which may be essential for receiving external signals, will prevent the GGDEF domains from forming active homodimers (Paul et al., 2007). Many DGCs and PDEs contain responsive regulator (REC) domains that can receive input signals for responding to environmental stimulation. A well-characterized *P. aeruginosa* strain contains several DGCs and PDEs that regulate cellular c-di-GMP levels and sense input signals, such as chemoattractants (WspR) and oxygen-deprived conditions (SadC), to alter intracellular c-di-GMP levels (O'Connor et al., 2012; Schmidt et al., 2016). For Gram-negative bacteria, the transmembrane GGDEF protein is generally located on the inner membrane and can form components of the response pathway with sensory proteins located in the periplasm or outer membrane (Kim and Harshey, 2016; Schmidt et al., 2016). However, the study of transmembrane GGDEF proteins is relatively poor in Gram-positive bacteria with only a single layer of membrane structure. For example, DgcK, a typical transmembrane DGC protein, has a synergistic effect with the degenerated GGDEF-transmembrane protein Ydak to regulate the production of an unknown EPS in Gram-positive *B. subtilis* (Bedrunka and Graumann, 2017b). There are five transmembrane helices in DgcA with a similar structure to DgcK, which belongs to the 5TMR-LYT family (5 transmembrane receptors of the LytS-YhcK type; PF07694), although they lack similarity in amino acid sequence alignment. However, the type of input signals that activate DgcK via 5TMR-LYT remain unknown.

We showed that DgcA is responsible for several phenotypes involved in biofilm formation, EPS synthesis, and co-aggregation in *L. acidophilus*. EPSs of probiotics are important in alleviating lactose intolerance, enhancing immunity against pathogens, and reducing mutagenic enzymes, such as β-glucuronidase, nitroreductase, and choloylglycine hydrolase (de Roos and Katan, 2000). In our experiments, DgcA expressed without the N-terminal hydrophobic sequence exhibited no activity *in vitro*, so we tried to prove the DGC activity by *in vivo* assay referred to a previous study (Chen et al., 2014; Purcell and Tamayo, 2016). The related results can be compared from the intracellular expression of pBAD vector and pBAD-*dgcA* (Figures 3, 6). Especially, when we *in vivo* expressed empty pMG36e vector and pMG36e-*dgcA* in La-14 respectively the EPS formation and co-aggregation are significantly higher (Figures 7, 8). All these functional tests proved that the DgcA has its activity *in vivo*. The functions of DgcA protein could be achieved *in vivo* by both through a c-di-GMP dependent (Chen et al., 2014) and independent (Holland et al., 2008) mechanism. On the other hand, the PDE activity of PdeA and the c-di-GMP receptor (PdeB) have been confirmed in assays *in vitro*. Combined with the evidence of *in vivo* assay, DgcA may be involved in c-di-GMP metabolism in *L. acidophilus*. Because of the concentration of c-di-GMP is hardly measured in the DgcA expressed bacterial lysate (containing complex components, data not shown) with HPLC used in our experiments, so the functions of DgcA could be achieved *in vivo* by both of the mechanisms which we will identify in the next step. Overexpression of PdeA resulted in changes in structure, but with no phenotypic changes observed in Congo red or co-aggregation assays. We hypothesized that this phenomenon was due to the low background concentration of intracellular c-di-GMP levels in *L. acidophilus* La-14 (only one copy of diguanylate cyclase gene in its genome).

It has been shown that *L. acidophilus* EPS is responsible for cell co-aggregation, which is an important characteristic of *Lactobacillus* that plays a critical role in its vitality (Goh and Klaenhammer, 2010). The operon *gts* encoded a BcsA-like glycosyltransferase (GtsB) and a hypothetical protein (GtsA) with double transmembrane loops, whose function appears to be involved in bacterial capsule biosynthesis, like cellulose or polymeric N-acetyl-glucosamine synthases and is associated with bacterial biofilm formation (Itoh et al., 2005; O'Gara, 2007; Morgan et al., 2013). Considering the above information, our data indicate that the genes *La14_RS07015* to *La14_RS07020* may be involved in *L. acidophilus* EPS formation through an unknown synthesis pathway (Figure 6). GtsB may serve as a poly-beta-1,6-N-acetyl-D-glucosamine or a catalytic subunit of poly-beta-1,4-D-glucopyranose synthase, while GtsA functions as synthase regulatory subunit. PdeB may bind to c-di-GMP to allosterically modulate enzymatic functions of GtsA/B through protein-protein interactions. The function of *gts* operon-encoded proteins in *L. acidophilus* may be similar to the Pss EPS synthase in *L. monocytogenes* or the cellulose synthase in *R. sphaeroides* (Omadjela et al., 2013; Chen et al., 2014; Köseoglu et al., 2015). Based on the references and our experiments, we speculate that c-di-GMP can bind with the PdeB and induce conformation changes through allosteric regulation of PdeB. This allosteric effect will remove the inhibitory interactions on the PdeB-GtsA/B complex or activate the idle state of GtsA/B to enhance the catalytic activity of GtsA/B (subunits A and B forming a glycosyltransferase) in EPS synthesis. The resulting production of EPS may increase the intercellular adhesion capacity of *L. acidophilus* and promote it to a higher aggregative state, both of which are characteristics of *L. acidophilus* as a probiotic. This allows *L. acidophilus* to colonize the host (oral cavity, gastrointestinal tract, and vagina) more easily and provides an advantage during bacterial competition in biofilms. Although the composition of *L. acidophilus* EPS remains unclear, we uncovered a potential regulatory pathway where input signals regulate *L. acidophilus* EPS production via intracellular DgcA and PdeA, allowing for physiological changes in the bacteria to cope with changes in the external environment.

Our study demonstrated that *L. acidophilus* might have a complete signaling system, regulating intracellular c-di-GMP levels, or a c-di-GMP-independent mechanism (depending on the direct evidence whether the DgcA could synthesize c-di-GMP to be got), both of which in turn could regulate EPS synthesis and coaggregation. However, some questions remain regarding c-di-GMP signaling in *Lactobacillus*, including whether the transmembrane protein DgcA actually synthesize c-di-GMP in *L. acidophilus* and how DgcA is involved in upstream signaling to control c-di-GMP synthesis, the composition of Gts EPS, and whether Gts EPS contributes to other phenotypes in *L. acidophilus*. Further studies should be conducted to better understand this process.

## Author contributions

JH designed and did the experiments with gene construction, culture experiments, biochemical tests, analyzed data, and wrote the manuscript. WR and JS did the experiments with biochemical tests. WY and FW provided overall directions and contributed to revising the manuscript.

### Conflict of interest statement

The authors declare that the research was conducted in the absence of any commercial or financial relationships that could be construed as a potential conflict of interest.

## Supplementary material

Figure S1 Multiple alignment results using a column-based method. The red color indicates highly conserved columns and the blue indicates less conserved ones. **(A)** Amino acid sequence alignment of conserved DgcA residues with other homologous proteins from *L. amylovorus, L. kalixensis, L. crispatus, L. frumenti, L. vaginalis, and L. reuteri*. **(B)** Amino acid sequence alignment of conserved PdeA residues with other homologous proteins from *L. amylovorus, L. crispatus, L. kalixensis, L. vaginalis, L. frumenti, and L. reuteri*.

Table S1 Primers used in the analysis of operon transcription.
