# Supplementary figures and images for: Functional Characterization of c-di-GMP Signaling-Related Genes in the Probiotic Lactobacillus acidophilus
Source: Front Microbiol. 2018 Aug 29;9:1935. doi: 10.3389/fmicb.2018.01935 (PMC6123363; doi:10.3389/fmicb.2018.01935)

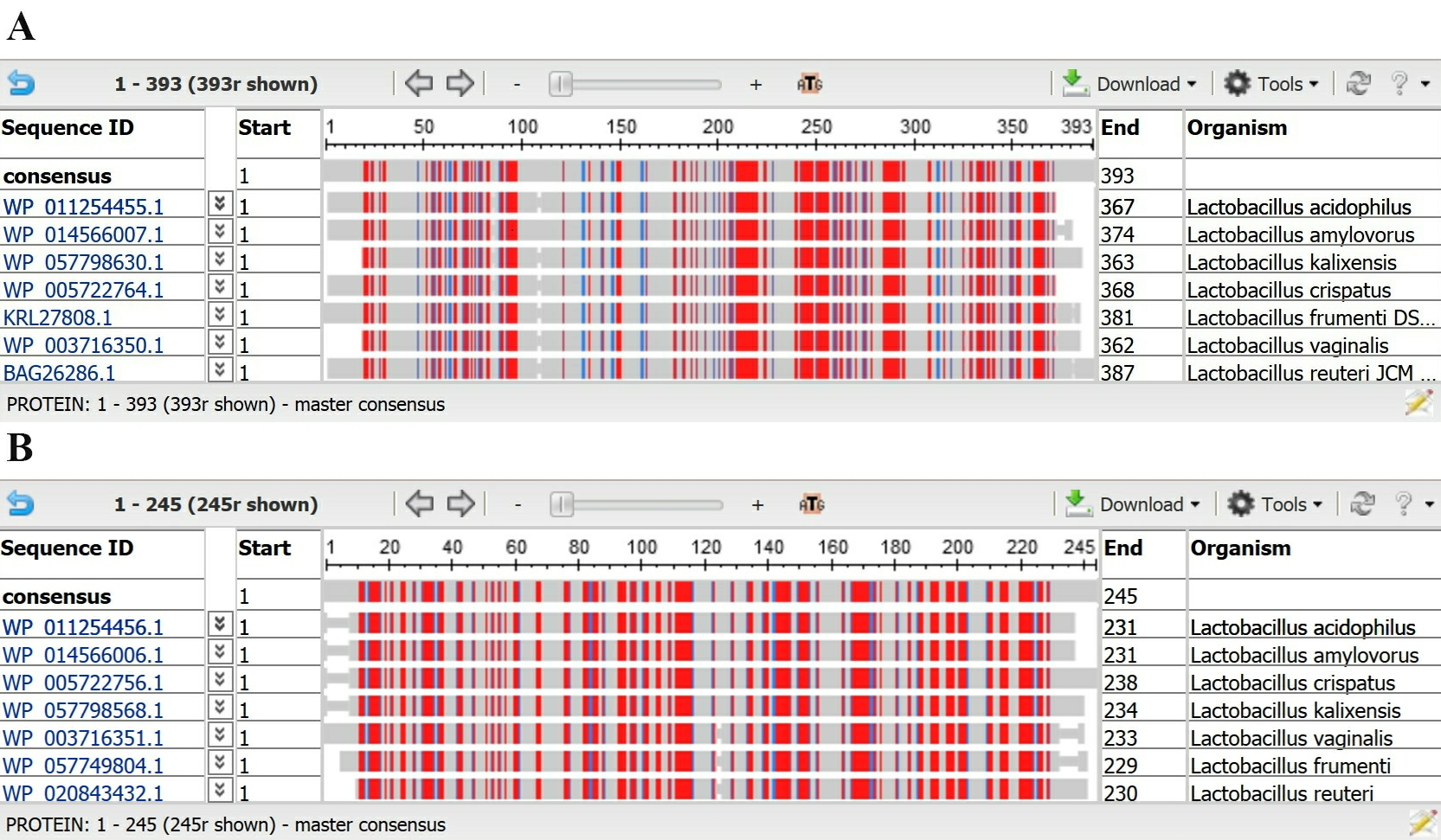

Supplement: Figure S1 — Multiple alignment results using a column-based method. The red color indicates highly conserved columns and the blue indicates less conserved ones. (A) Amino acid sequence alignment of conserved DgcA residues with other homologous proteins from L. amylovorus, L. kalixensis, L. crispatus, L. frumenti, L. vaginalis, and L. reuteri. (B) Amino acid sequence alignment of conserved PdeA residues with other homologous proteins from L. amylovorus, L. crispatus, L. kalixensis, L. vaginalis, L. frumenti, and L. reuteri. [file Image_1.jpg]
