# Supplementary material for: Functional Characterization of c-di-GMP Signaling-Related Genes in the Probiotic Lactobacillus acidophilus
Source: Front Microbiol. 2018 Aug 29;9:1935. doi: 10.3389/fmicb.2018.01935 (PMC6123363; doi:10.3389/fmicb.2018.01935)
Supplement: Table S1 — Primers used in the analysis of operon transcription. [file Table_1.docx]

| Premiers | Description | Reference |
| --- | --- | --- |
| Gene sequences A-F | CATCCCTTTTAATTTGGCCAA | This work |
| Gene sequences A-R | AAGCCATAATGTTCAAATTTAGTAGC | This work |
| Gene sequences B-F | GTTTACAAACCACCCTAAAAACC | This work |
| Gene sequences B-R | CAGGGGTCAGAGCAAAAAGG | This work |
| Gene sequences C-F | CCTAGTTTGTTGGTCTGTCTTAC | This work |
| Gene sequences C-R | ATCATGATTAGGGAATTGGTCG | This work |
| Gene sequences D-F | AACAAGCATATTTTCCAACAAGG | This work |
| Gene sequences D-R | CTAAGCGGAAGGTAGATTAATGG | This work |
| Gene sequences E-F | TGAACATAGATAAAGATGTCGAAGG | This work |
| Gene sequences E-R | TCTTCATTATGAGCCGGGACC | This work |
| Gene sequences F-F | GTCCCAAGGGTTTAAACTATGC | This work |
| Gene sequences F-R | CTTCATCTATGCCTGTTTTTTGTCG | This work |
| Gene sequences G-F | TGTTGGTGGTGTTGATAGTCC | This work |
| Gene sequences G-R | TCTTCATTATGAGCCGGGACC | This work |

Table S1 Primers used in the analysis of operon transcription
